# Dataset for a randomised factorial experiment to optimise an information leaflet for women with breast cancer

**DOI:** 10.3310/nihropenres.13547.1

**Published:** 2024-05-30

**Authors:** Sophie M C Green, Samuel G Smith

**Affiliations:** 1 Leeds Institute of Health Sciences, University of Leeds, Leeds, England, LS2 9LU, UK

**Keywords:** factorial, intervention optimisation, multiphase optimisation strategy, breast cancer, information leaflet

## Abstract

**Background:**

Adherence to adjuvant endocrine therapy (AET) is low in women with breast cancer, which increases the risk of recurrence and mortality. A consistently reported barrier to adherence is low perceived necessity of AET and high concerns. Existing interventions to support medication beliefs have mixed effectiveness and rarely target medication beliefs specifically. We developed an information leaflet with five candidate components aiming to increase necessity beliefs about AET and reduce concerns; (1) diagrams explaining how AET works; (2) icon arrays displaying the benefits of AET; (3) information about the prevalence of side-effects; (4) answers to common concerns and (5) quotes and pictures from breast cancer survivors. Guided by the multiphase optimisation strategy (MOST), we aimed to optimise the content of the information leaflet. We planned for the dataset to be open access to provide an exemplar for other investigators to use.

**Methods:**

The content of the leaflet was optimised in a fully powered online 2 ^5^ factorial experiment. Each candidate component of the leaflet was operationalised as a factor with two levels; on vs off or enhanced vs basic. Healthy women (n=1604) completed the beliefs about medicines questionnaire and were randomised to view one of 32 versions of the information leaflet. The 32 versions comprised unique combinations of the factor levels corresponding to the five candidate intervention components. Time spent on the information leaflet page of the survey was recorded. After viewing the information leaflet, participants completed the beliefs about medicines questionnaire again, a true/false questionnaire assessing their objective knowledge of AET, a subjective rating of their knowledge of AET, and a questionnaire evaluating their satisfaction with the information they received.

**Importance of this dataset:**

The factorial dataset provides the opportunity for other investigators interested in using the MOST framework to learn about complex factorial designs, using a real dataset.

## Plain Language Summary

Most women with breast cancer are treated with adjuvant endocrine therapy (AET) to reduce the chance of breast cancer coming back. However, many women do not take the medication as recommended. Women’s beliefs about the medication are a common reason for not taking AET. Some women do not think AET will help them, and some women have lots of concerns about AET. At the moment, we do not know the best way to change women’s beliefs about AET. Therefore, we ran a study to help us understand what combination of information might help change women’s beliefs about AET.

We developed a written information leaflet with five parts; (1) diagrams about how AET works; (2) visual figures of the benefits of AET; (3) information about how likely each side-effect is; (4) answers to common concerns about AET; and (5) pictures and quotes from women who have taken AET. In an online survey, 1,604 healthy women answered questions about their beliefs about the medication. Each woman was shown one version of the information leaflet picked at random. There were 32 possible versions of the information leaflet, which contained unique combinations of the five parts of the leaflet. After women read the leaflet, they were asked to complete the same questionnaire about their beliefs about the medication. They were also asked questions about how satisfied they were with the information they received, true or false questions about AET to assess their knowledge after reading the leaflet, and a rating of how informed they felt about AET. We also recorded how long women spent looking at the leaflet. One of our aims was to make the dataset from this experiment openly available so other scientists could use it to learn how to conduct similar experiments.

## Introduction

Adjuvant endocrine therapy (e.g., tamoxifen, anastrozole, letrozole, exemestane) reduces breast cancer recurrence and mortality in women with early-stage (I-III) breast cancer ^1, 2^. However, up to three-quarters of women do not take AET as prescribed ^3– 5^. A consistently cited barrier to AET adherence is medication beliefs. Low perceived necessity of AET and high concerns surrounding AET (e.g., concerns of potential side-effects) have been associated with lower adherence ^6– 10^.

Educational interventions are commonly used to address medication beliefs ^11^. However, evidence is mixed regarding the effectiveness of such interventions in changing medication beliefs ^11, 12^. Moreover, medication beliefs are often targeted within a larger complex intervention aiming to support AET adherence. Therefore, the effectiveness of components specifically targeting medication beliefs is unclear ^11, 12^.

To increase necessity beliefs and reduce concerns in women prescribed AET, we developed a theory-informed multicomponent educational information leaflet intervention ^13^. The information leaflet had five candidate components; (1) diagrams explaining how AET works; (2) icon arrays stating the benefits of AET; (3) side-effect prevalence information; (4) answers to common concerns; and (5) quotes and pictures from other breast cancer survivors ^13^.

Typically, an information leaflet intervention may be evaluated using a parallel group randomised controlled trial (RCT), comparing whether the leaflet as a whole is more effective than a suitable comparator, such as usual care. However, in a parallel group RCT, effects of the individual components of the leaflet, and interactions between intervention components cannot be estimated ^14^. It is possible some intervention components could be redundant, or could have a negative effect on beliefs about AET, meaning the effectiveness and efficiency of the leaflet may be compromised ^14^.

The multiphase optimisation strategy (MOST) is an engineering-inspired framework aiming to optimise complex interventions to balance effectiveness with efficiency ^14^. MOST proposes an optimisation phase prior to definitive evaluation of complex interventions. In the optimisation phase, highly efficient and fully powered experimental designs, such as factorial designs, can be used to estimate the individual and combined effects of intervention components ^14^. Empirical data from an optimisation trial can be used to select an optimised intervention ^15^; for example, selecting an intervention comprising only intervention components estimated to contribute to a positive effect on beliefs about AET. The MOST framework therefore offers the potential to balance intervention effectiveness with efficiency.

As the MOST framework is a novel approach to optimising and evaluating complex interventions, there are relatively few open access examples of data of optimisation trials. Open access datasets of factorial experiments would be useful to provide scientists learning about the framework with some real data to practice analysing and interpreting the data. Our aim was to create a dataset of our factorial experiment that is available for other investigators to use. The original aim of the factorial experiment leading to creation of this dataset was to optimise the content of an information leaflet intervention targeting beliefs about AET ^13^.

## Methods

### Patient and Public Involvement

A panel of four women prescribed AET for breast cancer were involved in this project. The panel were recruited in 2021 via a local charity supporting people affected by cancer. Through regular (approximately quarterly) meetings with the investigator(s), the panel had input into the design of the study (i.e., targeting medication beliefs), the targets of the intervention components and the content and design of the intervention components making up the information leaflet. They additionally provided quotes of their motivations for taking AET and advised on wording of the scenario presented at the beginning of the survey to provide context around being prescribed AET ^13, 16^. The panel did not contribute to the conduct or recruitment of the study. After further evaluation of the information leaflet in a larger trial ^17^, the panel will be consulted on methods of dissemination of study results.

### Ethical approval

Ethical approval was granted from the University of Leeds School of Medicine ethical review board (MREC 21-033, 21/03/2022). Written informed consent was obtained electronically from all participants. All procedures, including the informed consent process, were conducted in accordance with the ethical standards of the responsible committee on human experimentation (institutional and national) and with the Helsinki Declaration of 1975, as revised in 2000.

Full methods and a detailed description of the development of the candidate intervention components are described elsewhere ^13, 18^.

### Candidate intervention components

The information leaflet was made up of five candidate intervention components, each operationalised as factors with two levels: (1) diagrams explaining how AET works (levels: on/off); (2) icon arrays visually displaying the benefits of taking AET in terms of reduced recurrence and mortality (levels: enhanced/basic); (3) information about potential side-effects from AET and their prevalence (levels: enhanced/basic); (4) answers to common concerns women have about taking AET (levels: on/off); (5) quotes and pictures from breast cancer survivors, stating their motivations for taking AET (levels: on/off).

### Participants

Participants were required to be female, over 18 and able to read English. A market research company recruited participants via sending the survey link to potential respondents in the UK. A total of 1,604 women completed the survey. One participant was excluded due to being under 18.

### Design

We used a 2 ^5^ (2x2x2x2x2) factorial design ( Table 1). After completing basic demographic information, participants were shown a scenario asking them to imagine they had been diagnosed with breast cancer and prescribed AET. This scenario is available elsewhere ^13^. Participants could not proceed to the next page of the survey until 30 seconds had passed.

**Table 1.** Experimental conditions in 2 ^5^ factorial design and number randomized to each condition.

	Constant Component	Diagrams	Benefits	Side-effects	Common concerns	Patient input	Number randomised
1	Yes	Yes	Enhanced	Enhanced	Yes	Yes	55
2	Yes	Yes	Enhanced	Enhanced	Yes	No	54
3	Yes	Yes	Enhanced	Enhanced	No	Yes	53
4	Yes	Yes	Enhanced	Enhanced	No	No	38
5	Yes	Yes	Enhanced	Basic	Yes	Yes	53
6	Yes	Yes	Enhanced	Basic	Yes	No	56
7	Yes	Yes	Enhanced	Basic	No	Yes	47
8	Yes	Yes	Enhanced	Basic	No	No	58
9	Yes	Yes	Basic	Enhanced	Yes	Yes	45
10	Yes	Yes	Basic	Enhanced	Yes	No	57
11	Yes	Yes	Basic	Enhanced	No	Yes	42
12	Yes	Yes	Basic	Enhanced	No	No	50
13	Yes	Yes	Basic	Basic	Yes	Yes	54
14	Yes	Yes	Basic	Basic	Yes	No	41
15	Yes	Yes	Basic	Basic	No	Yes	49
16	Yes	Yes	Basic	Basic	No	No	63
17	Yes	No	Enhanced	Enhanced	Yes	Yes	45
18	Yes	No	Enhanced	Enhanced	Yes	No	55
19	Yes	No	Enhanced	Enhanced	No	Yes	56
20	Yes	No	Enhanced	Enhanced	No	No	42
21	Yes	No	Enhanced	Basic	Yes	Yes	61
22	Yes	No	Enhanced	Basic	Yes	No	52
23	Yes	No	Enhanced	Basic	No	Yes	54
24	Yes	No	Enhanced	Basic	No	No	58
25	Yes	No	Basic	Enhanced	Yes	Yes	44
26	Yes	No	Basic	Enhanced	Yes	No	51
27	Yes	No	Basic	Enhanced	No	Yes	40
28	Yes	No	Basic	Enhanced	No	No	50
29	Yes	No	Basic	Basic	Yes	Yes	46
30	Yes	No	Basic	Basic	Yes	No	39
31	Yes	No	Basic	Basic	No	Yes	43
32	Yes	No	Basic	Basic	No	No	52

*Note.* Each component had two levels: on vs off, or enhanced vs basic. This table was taken directly from Green *et al*., ^13^.

Participants completed the beliefs about medicines questionnaire. They were then randomised to one of 32 experimental conditions. Each condition corresponded to a unique version of the information leaflet made up of different combinations of the factor levels corresponding to the five intervention components. All possible combinations of factor levels made up the 32 experimental conditions. Participants could not proceed with the survey until three minutes had passed. After viewing the leaflet, participants were asked the same questions about their beliefs about AET, in addition to true or false questions about their knowledge of AET, their subjective knowledge of AET and their satisfaction with information they had received about AET. The questionnaires are available online (DOI 10.17605/OSF.IO/3CZ9Q) ^19^.

### Outcome measures

Participant demographics. Age, marital status, education level, ethnicity, menopausal status, previous breast cancer diagnoses and any close relations diagnosed with breast cancer were collected at the beginning of the survey. For women diagnosed with breast cancer, further questions about the stage and whether they had been prescribed AET were asked.

Beliefs about Medication Questionnaire-AET (BMQ-AET). The BMQ-AET is a 10-item scale used to assess specific medication beliefs ^20^. It is made up of two subscales; necessity and concerns, each with five items. Concern scores were subtracted from necessity scores to create a BMQ differential score. A BMQ differential score was calculated for the BMQ completed before and after viewing the information leaflet.

Satisfaction with information about Medicines (SIMS). A modified version of the SIMS was used ^21^. Seven of the original SIMS items were removed due to not being relevant. One item was added asking about the benefits of taking AET. Participants were asked to rate their satisfaction with 11 different aspects of information about AET on a 5-point scale; too much, about right, too little, none received or none needed. Responses of “about right” and “none needed” indicated satisfaction with information and scored 1, while all other responses indicated dissatisfaction and scored 0. A total score was calculated (0–11).

Objective knowledge. Eight true or false items assessed objective knowledge of AET, relating to the mechanisms, benefits and side-effects of AET. Items answered correctly were scored as 1, items answered incorrectly were scored as 0. A total knowledge score was calculated.

Subjective knowledge. One item assessed participants subjective knowledge of AET; “How informed do you feel about hormone therapy for women with breast cancer”. Participants answered on a 0 (not very well informed at all) to 10 (very well informed) subscale.

Engagement. To observe engagement with the information leaflet, the length of time participants spent on the information leaflet page of the survey was recorded.

### Statistical considerations

Missing data. There was no missing data as all survey items were mandatory. Any non-completed responses were not recorded.

Sample size. A sample size of 1,524 was required to detect an effect size of 0.15, with power of 0.9 and alpha set to 0.1. Alpha was set to 0.1 as a decision-priority approach was taken in which Type I and Type II errors are equally detrimental to selecting an optimised information leaflet ^15^. Assuming 5% of participants would be speed responders (not completing the survey correctly), we increased the sample size to 1,604. The ‘MOST’ package in R Studio version 4.2.0 was used to calculate sample size ^22, 23^.

### Data cleaning

All data cleaning was conducted using R Studio, in R Statistical Software version 4.2.0 ^23^. R packages used to clean the data included ‘tidyverse’ version 2.0.0 ^24^, ‘dplyr’ version 1.0.10 ^25^, ‘tibble’ version 3.2.1 ^26^ and ‘descr’ version 1.1.8 ^27^. Data cleaning involved four main steps: (1) renaming variables; (2) scoring the questionnaires; (3) effect coding the factors relating to the five intervention components (i.e., -1, +1); and (4) exclusion of participant that was under 18. The raw dataset and R code used to clean the data are available at https://doi.org/10.5518/1467 ^28^.

## Consent

Written informed consent was obtained electronically from all participants. All procedures, including the informed consent process, were conducted in accordance with the ethical standards of the responsible committee on human experimentation (institutional and national) and with the Helsinki Declaration of 1975, as revised in 2000.

## Data Availability

The dataset is available in a restricted access data repository. The data is restricted access due to data protection concerns; the data is not directly identifiable but could be viewed as indirectly identifiable. Ethical approval was granted to share the dataset open access in the University of Leeds data repository. All participants consented to their data to be stored in this way, and to be available for use in other research. Repository Name: Restricted Access Data Repository Leeds Request to access the data set can be made at: https://doi.org/10.5518/1467 ^28^ The dataset contains the following files: Raw data- the raw dataset prior to any data cleaning (csv file). Clean dataset- the dataset after data cleaning (csv file). R script for data cleaning- the code used to clean the raw dataset (R script). Data notes- explanation of the variables in the cleaned dataset (PDF). Open Science Framework: Extended data for ‘Dataset for a randomised factorial experiment to optimise an information leaflet for women with breast cancer’, https://doi.org/10.17605/OSF.IO/3CZ9Q ^19^ This project contains the following files: Information Leaflet Survey V1.1.pdf Information Leaflet PIS V1.2.pdf Data are available under a Creative Commons Attribution 4.0 International Licence (CC-BY 4.0).

## References

[1] Early Breast Cancer Trialists' Collaborative Group (EBCTCG), Davies C Godwin J : Relevance of breast cancer hormone receptors and other factors to the efficacy of adjuvant tamoxifen: patient-level meta-analysis of randomised trials. *Lancet.* 2011;378(9793):771–784. 10.1016/S0140-6736(11)60993-8 21802721 PMC3163848

[2] Early Breast Cancer Trialists Collaborative Group: Aromatase inhibitors versus tamoxifen in early breast cancer: patient-level meta-analysis of the randomised trials. *Lancet.* 2015;386(10001):1341–1352. 10.1016/S0140-6736(15)61074-1 26211827

[3] Hershman DL Kushi LH Shao T : Early discontinuation and nonadherence to adjuvant hormonal therapy in a cohort of 8,769 early-stage breast cancer patients. *J Clin Oncol.* 2010;28(27):4120–4128. 10.1200/JCO.2009.25.9655 20585090 PMC2953970

[4] Murphy CC Bartholomew LK Carpentier MY : Adherence to adjuvant hormonal therapy among breast cancer survivors in clinical practice: a systematic review. *Breast Cancer Res Treat.* 2012;134(2):459–478. 10.1007/s10549-012-2114-5 22689091 PMC3607286

[5] Huiart L Bouhnik AD Rey D : Early discontinuation of tamoxifen intake in younger women with breast cancer: is it time to rethink the way it is prescribed? *Eur J Cancer.* 2012;48(13):1939–1946. 10.1016/j.ejca.2012.03.004 22464016

[6] Toivonen KI Williamson TM Carlson LE : Potentially modifiable factors associated with adherence to Adjuvant Endocrine Therapy among breast cancer survivors: a systematic review. *Cancers (Basel).* 2020;13(1):107. 10.3390/cancers13010107 33561076 PMC7794693

[7] Moon Z Moss-Morris R Hunter MS : Barriers and facilitators of adjuvant hormone therapy adherence and persistence in women with breast cancer: a systematic review. *Patient Prefer Adherence.* 2017;11(1):305–322. 10.2147/PPA.S126651 28260867 PMC5328144

[8] Moon Z Moss-Morris R Hunter MS : Understanding tamoxifen adherence in women with breast cancer: a qualitative study. *Br J Health Psychol.* 2017;22(4):978–997. 10.1111/bjhp.12266 28850763

[9] Cahir C Dombrowski SU Kelly CM : Women’s experiences of hormonal therapy for breast cancer: exploring influences on medication-taking behaviour. *Support Care Cancer.* 2015;23(11):3115–3130. 10.1007/s00520-015-2685-x 25744290

[10] Cahir C Guinan E Dombrowski SU : Identifying the determinants of adjuvant hormonal therapy medication taking behaviour in women with stages I-III breast cancer: a systematic review and meta-analysis. *Patient Educ Couns.* 2015;98(12):1524–1539. 10.1016/j.pec.2015.05.013 26054455

[11] Bright EE Finkelstein LB Nealis MS : A systematic review and meta-analysis of interventions to promote Adjuvant Endocrine Therapy adherence among breast cancer survivors. *J Clin Oncol.* 2023;41(28):4548–4561. 10.1200/JCO.23.00697 37531593 PMC10553065

[12] Sheils E Tillett W James D : Changing medication-related beliefs: a systematic review and meta-analysis of randomized controlled trials. *Health Psychol.* 2024;43(3):155–170. 10.1037/hea0001316 37870789

[13] Green SMC Hall LH French DP : Optimization of an information leaflet to influence medication beliefs in women with breast cancer: a randomized factorial experiment. *Ann Behav Med.* 2023;57(11):988–1000. 10.1093/abm/kaad037 37494669 PMC10578395

[14] Collins LM : Optimization of behavioral, biobehavioral, and biomedical interventions: the Multiphase Optimization Strategy (MOST).Cham: Springer International Publishing,2018. 10.1007/978-3-319-72206-1

[15] Collins LM : The completion of the optimization phase.In: *Optimization of Behavioral, Biobehavioral, and Biomedical Interventions: The Multiphase Optimization Strategy (MOST).*L.M. Collins, Editor. Springer International Publishing: Cham,2018;227–266. 10.1007/978-3-319-72206-1_7

[16] Green SMC French DP Graham CD : Supporting Adjuvant Endocrine Therapy adherence in women with breast cancer: the development of a complex behavioural intervention using Intervention Mapping guided by the Multiphase Optimisation Strategy. *BMC Health Serv Res.* 2022;22(1): 1081. 10.1186/s12913-022-08243-4 36002831 PMC9404670

[17] Smith SG Green SMC Ellison R : Refining and Optimising a behavioural intervention to Support Endocrine Therapy Adherence (ROSETA) in UK women with breast cancer: protocol for a pilot fractional factorial trial. *BMJ Open.* 2023;13(2): e069971. 10.1136/bmjopen-2022-069971 36737093 PMC9900066

[18] Green SMC : Decision-making in the multiphase optimization strategy (MOST): Applying decision analysis for intervention value efficiency (DAIVE) to optimize an information leaflet to promote key antecedents of medication adherence. *Transl Behav Med.* ,2024. 10.1093/tbm/ibae029 PMC11282575 38795061

[19] Green SMC Smith SG : Optimisation of an information leaflet to support medication beliefs in women with breast cancer. Open Science Framework. 2024. 10.17605/OSF.IO/3CZ9Q PMC10578395 37494669

[20] Brett J Hulbert-Williams NJ Fenlon D : Psychometric properties of the Beliefs about Medicine Questionnaire-Adjuvant Endocrine Therapy (BMQ-AET) for women taking AETs following early-stage breast cancer. *Health Psychol Open.* 2017;4(2): 2055102917740469. 10.1177/2055102917740469 29379627 PMC5779943

[21] Horne R Hankins M Jenkins R : The Satisfaction with Information about Medicines Scale (SIMS): a new measurement tool for audit and research. *BMJ Quality & Safety.* 2001;10(3):135–140. 10.1136/qhc.0100135.. PMC1743429 11533420

[22] Collins LM Huang L Dziak J : MOST: Multiphase Optimization Strategy. R package version 0.1.2. 2022. Reference Source

[23] R Core Team: R: A language and environment for statistical computing.R Foundation for Statistical Computing: Vienna, Austria,2022. Reference Source

[24] Wickham H Averick M Bryan J : Welcome to the Tidyverse. *J Open Source Softw.* 2019;4(43): 1686. 10.21105/joss.01686

[25] Wickham H François R Henry L : _dplyr: A grammar of data manipulation_.. R package version 1.0.10. 2022. Reference Source

[26] Müller K Wickham H : tibble: Simple data frames. R package version 3.2.1. 2023. Reference Source

[27] Aquino J Enzmann D Schwartz M : descr: Descriptive statistics. R package version 1.1.8. 2023. Reference Source

[28] Green SMC Smith SG : Dataset for a randomised factorial experiment to optimise an information leaflet for women with breast cancer. 2024. 10.5518/1467 PMC11320031 39145099

