# Supplementary material for: Dataset for a randomised factorial experiment to optimise an information leaflet for women with breast cancer
Source: NIHR Open Res. 2024 May 30;4:32. [Version 1] doi: 10.3310/nihropenres.13547.1 (PMC11320031; doi:10.3310/nihropenres.13547.1)
Supplement: Peer review history

# Reviewer report: Reviewer response for version 1

**DOI:** 10.3310/nihropenres.14705.r32251

**Published:** 2024-07-16

**Authors:** Caroline Charlton

**Affiliations:** 1 Northumbria University, Newcastle upon Tyne, England, UK

Thank you for the opportunity to review this exciting article. This article is a great example of extending open science practices and knowledge sharing, outlining an interesting and novel methodology for intervention optimisation, and allowing for other researchers interested within intervention optimisation to utilise the shared dataset and learn more about the MOST framework.

 The rationale for this article is clearly outlined, and the reported work is clear, concise, and technically sound. The methods provide a good overview of how the dataset was created, with links provided to previously published resources to further aid understanding on the intervention design and the decisions made during this process. Outcomes measures are clearly stated, although I would be interested to know whether there were any controls/measures in places regarding the engagement outcome, as the length of time spent on the leaflet survey page may not be indicative of actual engagement (e.g., the participant may have done something else whilst waiting to be allowed to move forward in the survey). The dataset is presented with clear instructions on how to access it and what files are contained within the dataset.

 Overall, a great article which is important for sharing the novel methodology utilised. I would recommend this article to be indexed without any need for revision.

Are sufficient details of methods and materials provided to allow replication by others?

Yes

Is the rationale for creating the dataset(s) clearly described?

Yes

Are the datasets clearly presented in a useable and accessible format?

Yes

Are the protocols appropriate and is the work technically sound?

Yes

Reviewer Expertise:

Health literacy, cancer screening, intervention design and evaluation. I recently published a paper evaluating the NHS cervical screening leaflet.

I confirm that I have read this submission and believe that I have an appropriate level of expertise to confirm that it is of an acceptable scientific standard.

# Reviewer report: Reviewer response for version 1

**DOI:** 10.3310/nihropenres.14705.r32110

**Published:** 2024-07-13

**Authors:** Emily A. Walsh

**Affiliations:** 1 University of Miami, Coral Gables, Florida, USA

This article, *Dataset for a randomised factorial experiment to optimise an information leaflet for women with breast cancer*, summarized a study of 1604 health women who completed a single, brief leaflet-based intervention to promote knowledge of adjuvant endocrine therapy. Authors concisely described the methods and procedures of the study, which was based at the University of Leeds, and consisted of recruiting women via a paid market research company. This public dataset would offer open access to data for researchers to practice and examine questions related to a Multiphase Optimization Strategy, or MOST approach, which is a novel and compelling analytic strategy used to identify which components of an intervention offer the most effect. Authors note the lack of current public datasets available to analyze via a MOST approach, and thus, this dataset will provide meaningful and impactful opportunities to researchers practicing and utilizing this approach. Notably, authors recruited healthy women to partake in this study on knowledge of a cancer treatment, which may slightly impact generalizability of related findings of this dataset versus a patient sample of women with breast cancer. However, the benefits of this dataset outweigh this minor consideration, which should be stated in subsequent, related publications from this dataset.

Are sufficient details of methods and materials provided to allow replication by others?

Yes

Is the rationale for creating the dataset(s) clearly described?

Yes

Are the datasets clearly presented in a useable and accessible format?

Yes

Are the protocols appropriate and is the work technically sound?

Yes

Reviewer Expertise:

Psycho-oncology, clinical psychology, breast cancer, adjuvant endocrine therapy adherence and medication management

I confirm that I have read this submission and believe that I have an appropriate level of expertise to confirm that it is of an acceptable scientific standard.
